# Association of short-term particulate matter exposure with suicide death among major depressive disorder patients: a time-stratified case-crossover analysis

**DOI:** 10.1038/s41598-022-12421-z

**Published:** 2022-05-19

**Authors:** In Young Hwang, Daein Choi, Jihoon Andrew Kim, Seulggie Choi, Jooyoung Chang, Ae Jin Goo, Ahryoung Ko, Gyeongsil Lee, Kyae Hyung Kim, Joung Sik Son, Sang Min Park

**Affiliations:** 1 Department of Family Medicine, Seoul National University Hospital, Seoul, South Korea; 2 Department of Biomedical Sciences, Seoul National University Graduate School, Seoul, South Korea; 3 Department of Medicine, Mount Sinai Beth Israel, Icahn School of Medicine at Mount Sinai, New York, NY USA; 4 Department of Psychiatry, Yale University School of Medicine, New Haven, CT USA; 5 Department of Family Medicine, National Center for Mental Health, Seoul, South Korea; 6 Department of Public Health and Medical Services, Seoul National University Hospital, Seoul, South Korea; 7 Comprehensive Care Clinic, Public Healthcare Center, Seoul National University Hospital, Seoul, South Korea; 8 Department of Family Medicine, Korea University Guro Hospital, Seoul, South Korea

**Keywords:** Psychology, Environmental sciences

## Abstract

There is growing evidence that suggests a potential association between particulate matter (PM) and suicide. However, it is unclear that PM exposure and suicide death among major depressive disorder (MDD) patients, a high-risk group for suicide. We aimed to assess the effect of short-term exposure to PM on the risk of suicide in MDD patients who are at high risk for suicide. We investigated the risk of suicide among 922,062 newly-diagnosed MDD patients from 2004 to 2017 within the Korean National Health Insurance Service (NHIS) database. We identified 3,051 suicide cases from January 1, 2015, to December 31, 2017, within the death statistics database of the Korean National Statistical Office. PMs with aerodynamic diameter less than 2.5 μm (PM2.5), less than 10 μm (PM10), and 2.5–10 μm (PM2.5–10) were considered, which were provided from the National Ambient Air Monitoring System in South Korea. Time-stratified case-crossover analysis was performed to investigate the association of particulate matter exposure to suicide events. The risk of suicide was significantly high upon the high level of exposure to PM2.5, PM2.5–10 (coarse particle) and PM10 on lag 1 (*p* for trend < 0.05). Short-term exposure to a high level of PM was associated with an elevated risk for suicide among MDD patients. There is a clear dose–response relationship between short-term PM exposures with suicide death among MDD patients. This result will be used as an essential basis for consideration when establishing an air pollution alarm system for reducing adverse health outcomes by PM.

## Introduction

There has been a significant increase in suicide cases in recent decades, posing a severe public health problem worldwide^1,2^. Globally, it is the second leading cause of death among young adults and approximately 800,000 people suicide every year^1^. Various social and environmental factors affect suicide, including culture, gender, age, socioeconomic status^3,4^. The risk of suicide is even higher with underlying disease (i.e., HIV, cancer)^5,6^, especially with a psychiatric disorder^2,3,7^. A previous study has noted that patients with depression or other mood disorders have a 20-fold increased suicide risk compared to the general population. Meanwhile, depression is becoming increasingly burdensome, and the number of incident cases of depression worldwide increased from 172 million in 1990 to 258 million in 2017, representing an increase of 49.9%^8^. Considering this trend, identifying and managing risk factors affecting suicide in major depressive disorder (MDD) patients is crucial.

Previous studies have shown that long-term exposure to particulate matter (PM) might increase the risk of depression through chronic inflammation and brain structure changes^9,10^. Additional studies were done to evaluated the effect of short-term exposure to PM and noted that it aggravates several psychiatric symptoms, including suicide attempts, increases emergency center visits, and hospitalization^11,12^. Based on these findings, recent studies have investigated the association of short-term exposure to PM with suicide and reported an elevated risk for suicide in the general population^13–16^.

However, this association of PM short-term exposure and suicide has not been explored in the depressive population. Since MDD patients are at high risk for suicide and vulnerable to short-term mood swings that PM might aggravate, a further investigation among MDD patients is merited. Therefore, we aimed to assess the effect of short-term exposure to PM on the risk of suicide in newly diagnosed MDD patients in Korea, using the National Health Insurance Service (NHIS) database.

## Methods

### Study population

The study population was derived from the Korean NHIS database. The NHIS is a single-payer of the Korean healthcare system and provides universal health insurance for 97% of Korean citizens^17^. The NHIS collects all forms of claimed healthcare service data, which includes outpatient visits, hospital admissions, emergency department usages, and pharmaceutic drug prescriptions. The NHIS provides collected data for research purposes, and multiple epidemiologic studies using this data have demonstrated its validity^18^.

We enrolled 922,062 newly-diagnosed major depressive disorder (MDD) patients above 20 years old from the NHIS database who was diagnosed between January 1, 2004, to December 31, 2017. The diagnosis of MDD was defined with diagnosis codes of MDD (F32, F33) from the International Classification of Diseases, Tenth Revision (ICD‐10) and the use of anti-depressant medication at least once during the study period. Participants who were diagnosed with bipolar disease (ICD-10 code F31) and schizophrenia(ICD-10 code F20) were also excluded, because patient with both disease are often first diagnosed as depression, but they have different characteristics from patients with MDD. We followed the study participants from their initial diagnosis date of MDD until December 31, 2017.

Among 922,062 newly-diagnosed MDD patients, we identified 3085 suicide cases after January 1, 2015. Suicide events before January 1, 2015 were excluded in the main analysis due to the lack of PM2.5 data before 2015. The suicide events were defined by death due to intentional self-harm (ICD-10 codes X60–X84). 34 suicide cases were excluded from the analysis due to missing value for PM2.5 data and average daily temperature. Total 3051 suicide cases were included in the main analysis.

### Particulate matter exposure

The NHIS database also provides demographic information of the participants, which include the residential district code. Using the code, we have linked the residential district to the daily ambient level of PM10 and PM2.5, provided by the National Ambient Air Monitoring System in South Korea. There are approximately 300 atmospheric monitoring stations in Korea and these sites are selected according to certain criteria, such as the number of residents, location, emission source, and representative are of the target site. There was no district with more than two monitoring stations. All residential districts but two were covered by atmospheric monitoring stations, which covers 1.4% of the total population. Particulate matter levels in unmonitored sites were estimated by using the closest monitoring station^19^. The concentration of the coarse particle was calculated by subtraction of the PM2.5 value from that of PM10.

### Statistical analysis

We used a time-stratified case-crossover analysis study design to investigate the association of particulate matter exposure with suicide events. The case-crossover study is a validated study design to assess the short-term effect of the exposure. Each patient serves as his or her own control, thereby time-invariant individual variables such as age, sex, and individual comorbidities, are automatically controlled. Among several case-crossover designs, the time-stratified case-crossover design yielded better results with the least bias on previous systemic reviews^20,21^. We used single-day lag models to investigate the effect of PM exposure on suicide from lag0 (the day of the suicide event) to lag3 (3 days prior to the suicide event). Also, we used a 4-day cumulative lag model (lag0–3) to assess the effect of short-term cumulative PM exposure on suicidality. This is based on the results from the previous study, which noted that evidence of associations suggested short exposure periods lasting up to an average of 0–3 days^16^. The control days were matched by other days with the same day of the week from the same calendar month, and the same calendar year. The PM value of each case day (lag0 to lag3 and lag0–3) and control days were divided into approximate quartile. The conditional logistic regression was used to estimate the adjusted odds ratios (aORs) and 95% confidence intervals (CIs) of each quartile compared to the 1st quartile of the PM10, PM2.5, and coarse particles exposure, which represented the least exposure to the PM. An indicator variable for national holidays, an indicator variable for weekends, rainfall, and temperature were included as covariates. Stratified analysis according to subgroups of age, sex, household income, and duration of MDD, stratified analysis was conducted to examine the effect modification. According to the individual's physical activity and alcohol consumption, an additional stratified analysis was conducted on 1606 suicide cases among MDD patients who underwent health examinations within 2 years before the suicide event. All statistical tests were two-sided manner with a p value of less than 0.05. Data collection and statistical analyses were performed using SAS Enterprise Guide 7.1 (SAS Institute Inc., Cary, NC).

### Ethical consideration

This study was conducted in accordance with the Declaration of Helsinki and the study protocol approved by the Institutional Review Board (IRB) at the National Center for Mental Health, Seoul, Korea (IRB number: 116271-1027-57). The requirement for informed consent was waived by IRB at National Center for Mental Health since the patient information was de-identified and anonymized according to South Korean personal data protection laws.

## Results

The descriptive characteristics of the study population are depicted in Table 1 by chi-square test and analysis of variance test. Among 3051 suicide cases we have enrolled, 765, 767 759 and 760 cases were allocated for the first, second, third, and fourth quartile of lag0 PM10 exposure respectively. The mean PM10 values of the suicide day on each quartile were 23.6, 36.6, 48.9, and 78.7 μg/m^3^, respectively. Participants exposed to the higher concentration of PM10 on the day of suicide (lag0) were more likely to be exposed to high PM2.5 and coarse particle concentration. However, the distribution of age, gender, household income, disease duration, alcohol consumption and physical activity were not significantly different by quartiles of PM10 exposure.

**Table 1:** Descriptive characteristics of study population.

	PM10 1st quartile	PM10 2nd quartile	PM10 3rd quartile	PM10 4th quartile	*p* value
Suicide events	765	767	759	760	
**PM10 range (event day)**	5.5–30.8	30.9–42.1	42.2–56.3	56.5–558.9	
Lag0 PM10, µg/m^3^, mean (SD)	23.6 (5.5)	36.6 (3.2)	48.9 (4.0)	78.7 (44.0)	< 0.001
Lag0 PM2.5, µg/m^3^, mean (SD)	12.5 (4.3)	20.8 (4.7)	27.8 (6.2)	40.3 (13.6)	< 0.001
Lag0 PM2.5–10, µg/m^3^, mean (SD)	11.1 (3.5)	15.8 (4.2)	21.1 (6.0)	38.4 (40.7)	< 0.001
Age, years, mean (SD)	60.4 (14.9)	61.2 (15.5)	60.9 (14.5)	60.7 (14.4)	0.322
**Age, years, N (%)**					0.279
20–39	157 (20.5)	128 (16.7)	141 (18.6)	146 (19.2)	
≥ 40	608 (79.5)	639 (83.3)	618 (81.4)	614 (80.8)	
**Sex, N (%)**					0.137
Men	425 (55.6)	464 (60.5)	424 (55.9)	422 (55.5)	
Women	340 (44.4)	303 (39.5)	335 (44.1)	338 (44.5)	
**Household income, N (%)**					0.346
1st (highest)	230 (30.1)	266 (34.7)	226 (29.8)	231 (30.4)	
2nd	152 (19.9)	165 (21.5)	168 (22.1)	173 (22.8)	
3rd	159 (20.8)	150 (19.6)	160 (21.1)	160 (21.1)	
4th (lowest)	224 (29.3)	186 (24.3)	205 (27.0)	196 (25.8)	
**Disease duration, N (%)**					0.930
< 5 years	350 (45.8)	355 (46.3)	360 (47.4)	353 (46.5)	
≥ 5 years	415 (54.2)	412 (53.7)	399 (52.6)	407 (53.5)	
Participants who underwent health examinations	419	460	419	393	0.338
**Alcohol intake, N (%)**					
No	252 (60.1)	267 (58.0)	247 (58.9)	251 (63.9)	
Yes	167 (39.9)	193 (42.0)	172 (41.0)	142 (36.1)	
**Physical activity, N (%)**					0.610
No	225 (53.7)	253 (55.0)	238 (56.8)	228 (58.0)	
Yes	194 (46.3)	207 (45.0)	181 (43.2)	165 (42.0)	

*N* number of participants, *PM* particulate matter.

Table 2 shows the association between PM10 exposure and completed suicide events among MDD patients. Compared to those exposed to the lowest concentration of PM10 on lag1 (a day before the suicide event), those exposed to the highest concentration of PM10 on lag1 had higher odds of completed suicide (aOR 1.19, 95% CI 1.03–1.36). Furthermore, the risk of suicide increased upon the higher exposure to PM10 on lag1 (*p* for trend = 0.017).

**Table 2:** Association of PM10 exposure and suicide events among major depressive disorder patients.

	1st quartile	2nd quartile	3rd quartile	4th quartile	P for trend
**Lag0**					
PM10 range (µg/m^3^)	5.5–30.8	30.9–42.1	42.2–56.3	56.5–558.9	
Suicide events	765	767	759	760	
aOR (95% CI)	1.00 (reference)	1.00 (0.89–1.13)	0.96 (0.84–1.09)	0.95 (0.83–1.09)	0.405
**Lag1**					
Suicide events	728	759	776	788	
aOR (95% CI)	1.00 (reference)	1.10 (0.97–1.24)	1.14 (1.01–1.29)	1.19 (1.03–1.36)	0.017
**Lag2**					
Suicide events	729	783	780	759	
aOR (95% CI)	1.00 (reference)	1.12 (0.99–1.27)	1.11 (0.98–1.26)	1.08 (0.94–1.24)	0.383
**Lag3**					
Suicide events	746	747	809	749	
aOR (95% CI)	1.00 (reference)	0.99 (0.88–1.12)	1.07 (0.94–1.21)	0.97 (0.85–1.12)	0.973
**Lag0–3**					
Suicide events	765	764	739	783	
aOR (95% CI)	1.00 (reference)	0.99 (0.87–1.12)	0.93 (0.81–1.08)	1.01 (0.87–1.18)	0.977

Odds ratio estimated by conditional logistic regression adjusted for mean daily temperature, precipitation and holidays.

*PM* particulate matter, *aOR* adjusted odds ratios, *CI* confidence interval.

The association of PM2.5 exposure and suicide events on MDD patients is demonstrated in Table 3. Patients exposed to the highest level of PM2.5 on lag1 was associated with higher odds of suicide (aOR 1.17, 95% CI 1.03–1.34) compared to those exposed to the lowest concentration of PM2.5 on lag 1. The dose-responsive association of PM2.5 exposure and suicide was also statistically significant (*p* for trend 0.021).

**Table 3:** Association of PM2.5 exposure and suicide events among major depressive disorder patients.

	1st quartile	2nd quartile	3rd quartile	4th quartile	P for trend
**Lag0**					
Range (µg/m^3^)	0.0–16.1	16.1–23.1	23.1–32.0	32.0–113	
Suicide events	755	762	766	768	
aOR (95% CI)	1.00 (reference)	1.00 (0.89–1.12)	0.99 (0.88–1.12)	1.00 (0.87–1.14)	0.942
**Lag1**					
Suicide events	731	761	759	800	
aOR (95% CI)	1.00 (reference)	1.08 (0.96–1.21)	1.09 (0.96–1.23)	1.17 (1.03–1.34)	0.021
**Lag2**					
Suicide events	744	783	762	762	
aOR (95% CI)	1.00 (reference)	1.08 (0.96–1.21)	1.03 (0.91–1.17)	1.03 (0.91–1.17)	0.860
**Lag3**					
Suicide events	727	776	777	771	
aOR (95% CI)	1.00 (reference)	1.07 (0.96–1.21)	1.06 (0.94–1.20)	1.04 (0.91–1.18)	0.654
**Lag0–3**					
Suicide events	757	747	773	774	
aOR (95% CI)	1.00 (reference)	0.98 (0.87–1.11)	1.01 (0.89–1.15)	1.02 (0.89–1.17)	0.666

Odds ratio estimated by conditional logistic regression adjusted for mean daily temperature, precipitation and holidays.

*PM* particulate matter, *aOR* adjusted odds ratios, *CI* confidence interval.

The association of coarse particle exposure and suicide events is depicted in Table 4. There were statistically significant dose-responsive associations of completed suicide with coarse particle exposure on lag1 (*p* for trend 0.017). MDD patients who were exposed to the highest concentration of coarse particles on lag1 (4th quartile) had higher odds for suicide (aOR 1.19, 95% CI 1.03–1.38), compared to those who were exposed to the lowest concentration of coarse particle (1st Quartile).

**Table 4:** Association of coarse particle exposure and suicide events among major depressive disorder patients.

	1st quartile	2nd quartile	3rd quartile	4th quartile	P for trend
**Lag0**					
Range (µg/m^3)^	0.0–12.7	12.7–17.6	17.6–24.6	24.6–492.3	
Suicide events	755	769	756	771	
aOR (95% CI)	1.00 (reference)	1.02 (0.91–1.16)	1.01 (0.88–1.15)	1.03 (0.88–1.19)	0.812
**Lag1**					
Suicide events	738	751	773	789	
aOR (95% CI)	1.00 (reference)	1.06 (0.93–1.19)	1.12 (0.98–1.28)	1.19 (1.03–1.38)	0.017
**Lag2**					
Suicide events	760	759	759	773	
aOR (95% CI)	1.00 (reference)	1.01 (0.89–1.14)	1.00 (0.88–1.14)	1.04 (0.90–1.21)	0.634
**Lag3**					
Suicide events	739	759	774	779	
aOR (95% CI)	1.00 (reference)	1.03 (0.91–1.17)	1.06 (0.93–1.21)	1.08 (0.93–1.25)	0.303
**Lag0–3**					
Suicide events	744	780	748	779	
aOR (95% CI)	1.00 (reference)	1.08 (0.95–1.23)	1.03 (0.89–1.20)	1.11 (0.93–1.31)	0.365

Odds ratio estimated by conditional logistic regression adjusted for mean daily temperature, precipitation and holidays.

*PM* particulate matter, *aOR* adjusted odds ratios, *CI* confidence interval.

Table 5 shows the results of the stratified analysis on the association of PM exposure and suicidal events. The risk elevating effect of PM was prominent among MDD patients aged 40 years or older, women, within 5 years of MDD diagnosis, who do not exercise. Although the statistical significance tended to be attenuated most likely due to the reduce number of cases upon stratification, exposure to high concentration of PM on lag1 and had tendency to increase the risk for suicide among MDD patients in multiple subgroups.

**Table 5:** Association of PM exposure and suicide events among major depressive disorder patients according to subgroups.

	1st quartile [aOR (95% CI)]	2nd quartile [aOR (95% CI)]	3rd quartile [aOR (95% CI)]	4th quartile [aOR (95% CI)]	P for trend
**Age**					
**20–39**					
PM10 Lag1	1.00 (reference)	0.86 (0.64–1.14)	0.95 (0.71–1.28)	1.09 (0.80–1.50)	0.434
PM2.5 Lag1	1.00 (reference)	1.10 (0.83–1.45)	1.00 (0.75–1.33)	1.21 (0.89–1.63)	0.606
Coarse Lag1	1.00 (reference)	1.13 (0.85–1.50)	1.03 (0.75–1.40)	1.27 (0.90–1.78)	0.264
**≥ 40**					
PM10 Lag1	1.00 (reference)	1.16 (1.01–1.32)	1.18 (1.03–1.37)	1.20 (1.03–1.41)	0.024
PM2.5 Lag1	1.00 (reference)	1.07 (0.94–1.22)	1.11 (0.96–1.27)	1.16 (1.01–1.34)	0.042
Coarse Lag1	1.00 (reference)	1.04 (0.91–1.19)	1.14 (0.98–1.32)	1.17 (0.99–1.38)	0.036
**Sex**					
**Men**					
PM10 Lag1	1.00 (reference)	1.09 (0.93–1.28)	1.16 (0.98–1.37)	1.12 (0.93–1.35)	0.179
PM2.5 Lag1	1.00 (reference)	1.04 (0.89–1.22)	1.10 (0.93–1.29)	1.11 (0.94–1.33)	0.181
Coarse Lag1	1.00 (reference)	1.00 (0.85–1.17)	1.12 (0.94–1.34)	1.12 (0.92–1.36)	0.419
**Women**					
PM10 Lag1	1.00 (reference)	1.11 (0.92–1.33)	1.11 (0.91–1.36)	1.27 (1.03–1.57)	0.036
PM2.5 Lag1	1.00 (reference)	1.13 (0.94–1.35)	1.07 (0.89–1.29)	1.25 (1.03–1.52)	0.292
Coarse Lag1	1.00 (reference)	1.14 (0.94–1.37)	1.12 (0.91–1.37)	1.30 (1.03–1.64)	0.044
**Income**					
**High**					
PM10 Lag1	1.00 (reference)	1.10 (0.93–1.30)	1.14 (0.96–1.36)	1.19 (0.99–1.44)	0.072
PM2.5 Lag1	1.00 (reference)	1.10 (0.93–1.29)	1.08 (0.91–1.28)	1.15 (0.96–1.37)	0.175
Coarse Lag1	1.00 (reference)	1.02 (0.96–1.21)	1.16 (0.97–1.39)	1.16 (0.95–1.43)	0.079
**Low**					
PM10 Lag1	1.00 (reference)	1.10 (0.92–1.31)	1.13 (0.94–1.37)	1.18 (0.96–1.44)	0.123
PM2.5 Lag1	1.00 (reference)	1.06 (0.89–1.26)	1.10 (0.92–1.31)	1.19 (0.98–1.44)	0.071
Coarse Lag1	1.00 (reference)	1.09 (0.91–1.30)	1.06 (0.88–1.30)	1.23 (0.99–1.53)	0.101
**Disease duration**					
**< 5 years**					
PM10 Lag1	1.00 (reference)	1.31 (1.09–1.57)	1.31 (1.08–1.58)	1.28 (1.04–1.57)	0.036
PM2.5 Lag1	1.00 (reference)	1.26 (1.06–1.50)	1.23 (1.02–1.48)	1.21 (1.00–1.47)	0.099
Coarse Lag1	1.00 (reference)	1.17 (0.97–1.40)	1.26 (1.03–1.53)	1.27 (1.02–1.58)	0.028
**≥ 5 years**					
PM10 Lag1	1.00 (reference)	0.95 (0.81–1.12)	1.02 (0.86–1.21)	1.12 (0.93–1.35)	0.182
PM2.5 Lag1	1.00 (reference)	0.95 (0.81–1.12)	0.99 (0.84–1.17)	1.16 (0.97–1.38)	0.103
Coarse Lag1	1.00 (reference)	0.97 (0.82–1.14)	1.01 (0.85–1.21)	1.13 (0.92–1.38)	0.247
**Alcohol**					
**No**					
PM10 Lag1	1.00 (reference)	1.23 (0.99–1.52)	1.22 (0.98–1.53)	1.30 (1.02–1.66)	0.053
PM2.5 Lag1	1.00 (reference)	1.15 (0.94–1.42)	1.24 (1.01–1.54)	1.30 (1.04–1.64)	0.016
Coarse Lag1	1.00 (reference)	1.03 (0.83–1.27)	1.16 (0.92–1.46)	1.13 (0.87–1.46)	0.252
**Yes**					
PM10 Lag1	1.00 (reference)	1.06 (0.93–1.37)	1.20 (0.92–1.58)	1.27 (0.94–1.70)	0.082
PM2.5 Lag1	1.00 (reference)	1.15 (0.90–1.48)	1.21 (0.93–1.57)	1.23 (0.92–1.63)	0.154
Coarse Lag1	1.00 (reference)	1.00 (0.77–1.30)	1.22 (0.93–1.61)	1.30 (0.95–1.78)	0.054
**Exercise**					
**No**					
PM10 Lag1	1.00 (reference)	1.24 (1.00–1.54)	1.33 (1.05–1.69)	1.42 (1.10–1.83)	0.008
PM2.5 Lag1	1.00 (reference)	1.22 (0.98–1.52)	1.39 (1.11–1.74)	1.48 (1.17–1.88)	0.001
Coarse Lag1	1.00 (reference)	1.02 (0.82–1.28)	1.27 (1.00–1.61)	1.16 (0.89–1.52)	0.127
**Yes**					
PM10 Lag1	1.00 (reference)	1.06 (0.84–1.35)	1.07 (0.83–1.39)	1.13 (0.86–1.50)	0.394
PM2.5 Lag1	1.00 (reference)	1.07 (0.84–1.35)	1.04 (0.81–1.34)	1.04 (0.80–1.36)	0.815
Coarse Lag1	1.00 (reference)	1.01 (0.79–1.28)	1.09 (0.84–1.42)	1.25 (0.92–1.69)	0.140

Odds ratio estimated by conditional logistic regression adjusted for mean daily temperature, precipitation and holidays.

*PM* particulate matter, *N* number of participants, *aOR* adjusted odds ratios, *CI* confidence interval.

Supplementary Table 1 shows the suicide risk per interquartile range (IQR) increase in PM10, PM2.5, and coarse particle based on linear modeling of PM with suicide. There was a significant increased risk for suicide upon IQR increase in PM10 (aOR 1.05, 95% CI 1.01–1.10), and coarse particle (aOR 1.04, 95% CI 1.01–1.07) on lag1, but not in PM2.5.

## Discussion

In this study of 3051 suicide cases from 922,062 newly diagnosed MDD patients in South Korea, we found that short-term exposure to a high PM level was associated with increased suicide risk. The higher the concentration of PM (lag1) was, the higher the risk for completed suicide MDD patients had (p for trend < 0.05), confirming a dose-responsive relationship between PM level and risk of completed suicide. To our knowledge, this is the first and largest epidemiological study to assess suicide risk among MDD patients to PM exposure. We found a significant association between suicide risk and PM.

A recent study on 134,811 suicide cases in 10 cities in northeast Asia reported significantly increased suicidal risk upon the increased level of PM10 and coarse particles at lag 0–1 but not with PM2.5^16^. Similar findings were noted in multiple previous studies among the general population, although the results were slightly different from^13,14^. A study on 4341 suicide cases in 2004 in South Korea reported a significantly increased suicidal risk upon the increased level of both PM 10 at lag 0–2 and PM 2.5 at lag 1^13^, while another study on 1546 suicide cases from 2001 to 2010 in Utah USA reported an increased suicide risk associated with PM 2.5 levels at lag 2 but not with PM10^14^. Our study shows similar results with these studies, supporting increased suicidal risk upon the increased level of the PM, and further expands the concept to depression patients at high risk for suicide. Also, there was a dose-responsive elevated risk for suicide upon PM exposure in our study, showing an 19%, 17% and 19% increase risk for suicide among participants exposed to the highest level of PM10, PM2.5, and coarse particles respectively, compared to the least exposed group. Previous studies on the general population used a different measure of exposure such as an increase in interquartile range^13–16^. These studies reported 2–9% increased risk for suicide per interquartile range increase of PM level. Direct comparison of the strength of association is difficult with previous studies, however the result from our study was similar to the previous studies in general population.

PM exposure was associated with an increase in suicide, and various mechanisms have been suggested to explain this association, with the etiology of depression. The significant effects of PM are explained through low-grade systemic inflammation originating in peripheral tissues such as the lung and skin. Systemic-induced cytokines circulate the body and possibly causing neuroinflammation, neuronal damage, and neurotransmitter change. Since smaller particles enter the systemic circulation and invade the brain parenchyma more easily, PM2.5 and ultrafine PM (diameter < 0.1 μm) are usually considered as the leading cause of inflammatory damage from the PM^9^. Moreover coarse particles are known to cause more inflammatory reactions in an acute exposure^22,23^, sometimes immediate and excessive inflammatory reactions such as allergic reactions^24^. Beside chemical components on coarse PM enter through the olfactory mucosa and bulb affect the brain rapidly and directly, coarse particles might also be a major cause of short-term neuroinflammatory. This exacerbation of neuroinflammation by PM may aggravate the depressive symptoms and stimulate the hyperactivated hypothalamic–pituitary–adrenal axis, which might cause mood instability and future suicide risk.

Moreover, PM can affect mood swings and inadequate control of impulsivity in another way. Previous studies reported that PM could cause circadian rhythm disturbance by reducing sunlight or solar radiation and also affects mood swings^25,26^. This association can be explained by decreased serotonin activity. Serotonin is a crucial neurotransmitter in stabilizing mood and regulating aggression and impulsivity^27^ and is produced according to sunlight exposure. Since PM decreases sunlight exposure, it might reduce serotonin level and possibly cause aggressiveness or uncontrolled impulsivity, directly linked to completed suicide. Particularly serotonin's rapid turnover is especially crucial in the pathophysiology of MDD, so the impact due to serotonin reduction would be critical for MDD patients.

Increasing suicide risk on PM exposure can be understood with the stress–diathesis model. The stress–diathesis model is one of the explanatory and predictive models of suicidal behavior^28^. It is hypothesized that individuals with a diathesis (predisposes vulnerability to the stressor) will lead to suicidal behaviors when confronted with stressors. Acute psychiatric disease aggravation or psychosocial crises are a common stressor. Various factors such as sex, religion, genetic factor, childhood experiences, familial factors, and cholesterol levels influence the diathesis^28^. Higher PM is associated with increasing suicide by exacerbating the disease^13^, and PM is proposed as a novel environmental trigger of suicide^29^. Once depressive patients who already have a hypersensitive hypothalamic–pituitary–adrenal axis, which induces mood instability and impulsivity, are triggered by stressors such as PM can develop suicidal behavior readily.

In our study, a high PM concentration increased the risk of suicide among MDD patients in multiple subgroups. Although statistical significance declined due to the decrease in the number of samples, we need to consider that this result may be reflecting the subgroup characteristics of depressed patients who are sensitive to PM exposure. Participants aged 40 years or older, women, within 5 years of MDD diagnosis and who do not exercise were particularly susceptible to PM exposure (Table 5). Consistent with previous findings^13^, we found a strong association among participants aged over 40 years. It is probably because the middle age group (35–64 years old) is exposed to higher PM more often during commuting or other activity, and also elderly people are sensitive to PM exposure^30^. Women were strong associated with PM exposure in our study. This result is also similar to the previous study^31^, explained by estrogen’s proinflammatory effect while androgen has immunosuppressive when exposed PM^32^. Exercise and physical activity have beneficial effects as antidepressant^33^, lowers suicidal ideation^34^, which explains why people who do not exercise was sensitive to PM exposure on our study. Finally, the risk of suicide is highest within 90 days of diagnosis of depression depression (aOR 7.33, 95% CI 4.76–11.3)^35^ or the first 90 days after hospital discharge^36^. Moreover regardless of the severity or duration, patient with MDD has highest risk of suicide within 5 years of the diagnosis^37^, which explains the result of our study. The synergistic effect of predisposing factors of suicide and PM on mood swings could explain these results.

Our study has several limitations. First, suicide cases might have been underreported because it can be recorded as accidental or undetermined. Second, the participants' residence area might be different from the actual place where the participants spend most of the time, such as the workplace or school. The level of exposure to PM might not be accurately calculated in such cases. Further studies with a more accurate measure of an individual's exposure to PM will be needed. Third, the possible confounder, daylight hours, were not considered in the analysis. On the other hand, daylight hours vary over a seasonal time frame. Therefore, daylight hours of control dates within a few weeks from the case dates would not vary from the case daylight hours. Additionally, we tried to take account of meteorological effects including temperature and precipitation, which indirectly reflect the sunlights hours besides seasonal variation. Fourth, other underlying diseases that could be affected the risk for suicide were not considered. Although, we used a time-stratified case-crossover design, in which each participant serves as their control. However, it is estimated that exacerbation of underlying disease caused by PM exposure increases the risk of suicide, so it is necessary to investigate disease groups that are particularly sensitive to PM exposure. Fifth, the severity of the disease among MDD participants was not reflected. Drug compliance and symptoms of depression at the time of suicide were not accounted for. Therefore, further studies considering the severity of psychiatric symptoms and treatment regimen will be merited. Finally, the suicide date in our study was based on death registry data, so it is possible directly reflect the suicide attempted date. For example, if a participant has attempted suicide but died in the hospital a couple of days later, the recorded suicide date would be later than the exposure day. However, the 4-day cumulative lag model takes account for this possibility and suggests that short-term exposure to the high level of PM in MDD patients increases the risk for suicide.

Despite these limitations, this study was the first large-scale study investigating PM's effects on suicide among MDD patients. Conjunction with the etiology of depression, we tried to elucidate the effects of PM exposure on suicide in depressed patients.

## Conclusion

Short-term exposure to PM was associated with increased risk of suicide in pre-existing depressed patients. The evidence supporting the mechanism of PM on depression and mood swings were not enough. However, this result suggests that awareness of harm for PM is needed for public mental health, emphasizes the importance of establishing an air pollution alarm system. Further researches on PM's neurophysiological responses are needed to understand the potential mechanism of PM's impact on suicide.

## Supplementary Information

Supplementary Table 1.
