# Supplementary material for: Association of short-term particulate matter exposure with suicide death among major depressive disorder patients: a time-stratified case-crossover analysis
Source: Sci Rep. 2022 May 19;12:8471. doi: 10.1038/s41598-022-12421-z (PMC9120445; doi:10.1038/s41598-022-12421-z)
Supplement: Supplementary file 1 — Supplementary Table 1. [file 41598_2022_12421_MOESM1_ESM.docx]

**Supplementary Table 1. Association of PM exposure and suicide events among major depressive disorder patients.**

|  | **aOR (95% CI) per IQR increase** |
| --- | --- |
| **PM10** |  |
| Lag0 | 1.00 (0.96-1.05) |
| Lag1 | 1.05 (1.01-1.10) |
| Lag2 | 1.05 (1.00-1.10) |
| Lag3 | 1.02 (0.97-1.07) |
| Lag0-3 | 1.06 (1.00-1.12) |
| **PM2.5** |  |
| Lag0 | 0.99 (0.93-1.05) |
| Lag1 | 1.02 (0.97-1.08) |
| Lag2 | 1.01 (0.95-1.07) |
| Lag3 | 0.99 (0.94-1.05) |
| Lag0-3 | 1.01 (0.95-1.07) |
| **Coarse particle** |  |
| Lag0 | 1.01 (0.98-1.04) |
| Lag1 | 1.04 (1.01-1.07) |
| Lag2 | 1.04 (1.01-1.07) |
| Lag3 | 1.02 (0.99-1.05) |
| Lag0-3 | 1.05 (1.01-1.09) |

Odds ratio estimated by conditional logistic regression adjusted for mean daily temperature, precipitation and holidays.

Acronym: PM, particulate matter; aOR, adjusted odds ratios; CI, confidence interval; IQR, interquartile range
